# Ontological representation, integration, and analysis of LINCS cell line cells and their cellular responses

**DOI:** 10.1186/s12859-017-1981-5

**Published:** 2017-12-21

**Authors:** Edison Ong, Jiangan Xie, Zhaohui Ni, Qingping Liu, Sirarat Sarntivijai, Yu Lin, Daniel Cooper, Raymond Terryn, Vasileios Stathias, Caty Chung, Stephan Schürer, Yongqun He

**Affiliations:** 1 Department of Computational Medicine and Bioinformatics, University of Michigan, Ann Arbor, MI USA; 2 Unit of Laboratory Animal Medicine and Department of Micro biology and Immunology, University of Michigan, Ann Arbor, MI USA; 3 Samples, Phenotypes and Ontologies Team, European Bioinformatics Institute (EMBL-EBI), European Molecular Biology Laboratory, Hinxton, Cambridge, UK; 4 Department of Molecular and Cellular Pharmacology, University of Miami, Miami, FL USA; 5 BD2K LINCS Data Coordination and Integration Center, University of Miami, Miami, FL USA; 6 Center for Computational Science, University of Miami, Miami, FL USA

**Keywords:** Cell line, Lincs, Data integration, Ontology, Cell line ontology, ChEMBL

## Abstract

**Background:**

Aiming to understand cellular responses to different perturbations, the NIH Common Fund Library of Integrated Network-based Cellular Signatures (LINCS) program involves many institutes and laboratories working on over a thousand cell lines. The community-based Cell Line Ontology (CLO) is selected as the default ontology for LINCS cell line representation and integration.

**Results:**

CLO has consistently represented all 1097 LINCS cell lines and included information extracted from the LINCS Data Portal and ChEMBL. Using MCF 10A cell line cells as an example, we demonstrated how to ontologically model LINCS cellular signatures such as their non-tumorigenic epithelial cell type, three-dimensional growth, latrunculin-A-induced actin depolymerization and apoptosis, and cell line transfection. A CLO subset view of LINCS cell lines, named LINCS-CLOview, was generated to support systematic LINCS cell line analysis and queries. In summary, LINCS cell lines are currently associated with 43 cell types, 131 tissues and organs, and 121 cancer types. The LINCS-CLO view information can be queried using SPARQL scripts.

**Conclusions:**

CLO was used to support ontological representation, integration, and analysis of over a thousand LINCS cell line cells and their cellular responses.

## Background

Since immortalized cell lines were developed almost one century ago, various cell lines have been widely used to study various scientific biological and biomedical questions [1]. The NIH Common Fund Library of Integrated Network-based Cellular Signatures (LINCS) program [2] aims to create a network-based biological understanding of gene expression profiles and cellular processes when cells, mostly cell line cells, are exposed to various experimental conditions and perturbing agents (http://www.lincsproject.org/). Diverse, multi-dimensional datasets have been generated by LINCS groups and laboratories and used to generate extensive results and software programs. A major challenge is how to integrate large amounts of LINCS-generated data into a comprehensive integrative understanding of cellular signatures [3]. Given that cell line cells play a critical role in LINCS studies, it is possible to use cell line cells as a hub pointing to semantics link and integrate various molecular and cellular signatures and networks to address various biomedical questions.

Co-developed by many groups and societies, including the Cell Type Ontology (CL) [3], Ontology for Biomedical Investigations (OBI) [4], BioAssay Ontology (BAO) [5], Drug Target Ontology (DTO) [6], Vaccine Ontology (VO) [6, 7], the European Bioinformatics Institute (EBI), and the Japan Riken BioResource Center, the community-based Cell Line Ontology (CLO) [8] aims to comprehensively represent cell line cells, cell lines, and their related entities such as corresponding cell types, tissues, organs, organisms, and possible diseases. CLO is developed by following the principles of the Open Biological and Biomedical Ontologies (OBO) Foundry [9]. Currently, CLO represents nearly 40,000 cell lines from various resources such as the American Type Culture Collection (ATCC) (http://www.atcc.org/), HyperCLDB [10], Coriell Cell lines (https://catalog.coriell.org/), and Japan Riken cell lines (http://cell.brc.riken.jp/en/). CLO has been used in many projects such as BAO development [5], Beta cell genomes [11], Chromosome-Centric Human Proteome Project [12], ChEMBL database [13], and Cellosaurus (http://web.expasy.org/cellosaurus/). Among these resources, the ChEMBL database, a large-scale bioactivity database developed by the EBI [13, 14], is for drug-like compounds and contains many cell lines and their cross-references in different resources including CLO and LINCS.

In this study, we report our work on updating CLO to include all LINCS cell lines and additional information required by LINCS projects. Such a comprehensive and integrative representation makes CLO able to coherently represent and study all LINCS cell lines together. To support LINCS integrated study of cellular features and signatures, we have also updated CLO to include additional design patterns using the cell line model MCF 10A cell line cells.

## Methods

**Fig. 1:**
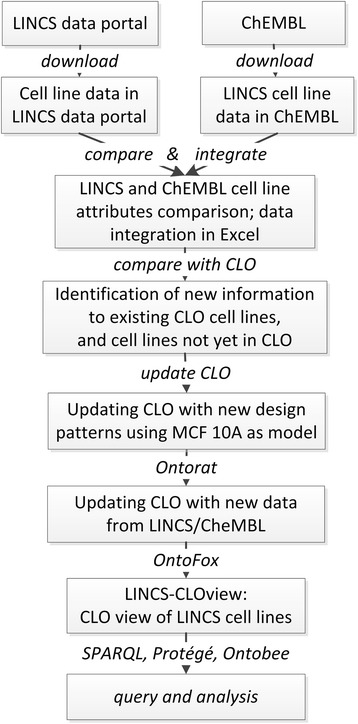
Project pipeline of integrating cell lines from LINCS data portal and ChEMBL into CLO

The overall workflow of this project (Fig. 1) includes and integrates different methods of this study. Specific methods of this pipeline are described below.

### CLO modeling of LINCS cell line information and design pattern generation

Based on the data types obtained from the mapping process, an updated CLO design pattern model was generated in order to accommodate new LINCS cell line data attributes.

### Information extraction of LINCS cell lines

Two sources, including the LINCS Data Portal (http://lincsportal.ccs.miami.edu/) [14] and ChEMBL, were used to obtain LINCS cell line information. The information of the cell lines available from the LINCS Data Portal was directly downloaded from its website. In our study, the ChEMBL data (release 21) was downloaded into a local MySQL database. LINCS cell lines available in the database were identified using LINCS_ID, and related data were then extracted from ChEMBL.

### Cell line data mapping and comparison between resources

Cell line-related data types or attributes were first compared between LINCS Data Portal and ChEMBL and then compared with the CLO knowledge base. To identify if a LINCS cell line found from the LINCS Data Portal and ChEMBL also exists in CLO, SPARQL scripts [15] were generated to map the labels of LINCS cell lines with CLO cell line labels or alternative names (i.e., cell line synonyms) using string-based name matching.

The mapped cell line data from CLO was also used to support LINCS cell line data integration. Following SPARQL-based identification of cell line names and synonyms within CLO, along with the corresponding Disease Ontology Identifiers (DOIDs), information for the LINCS cell lines was manually validated and curated to ensure accurate information matches. If existed, multiple members of the same cell line name or synonym were consolidated into a singular LINCS cell identifier.

### New information incorporation into CLO

Based on the new design patterns, Ontorat [16] was used to incorporate LINCS cell line data from different data sources to CLO. Two steps were performed. The first step was to add to existing CLO cell lines with new data obtained from LINCS and ChEMBL, and the second step was to add new LINCS cell lines to CLO. Manual checking was performed to ensure correctness.

### Generation of a LINCS cell line set of CLO

OntoFox [17] was used to generate a CLO subset (named as LINCS-CLOview) that includes all LINCS cell lines and related ontology terms and relations. The source code of the LINCS-CLOview was submitted to the CLO GitHub website with the following web page URL: https://raw.githubusercontent.com/CLO-ontology/CLO/master/src/ontology/LINCS-CLOview.owl. The LINCS CLO subset was also submitted to Ontobee [18]. The information of the subset can be queried using the Ontobee SPARQL web program (http://www.ontobee.org/sparql).

### CLO-based analysis of LINCS cell lines

Note that CLO and LINCS-CLOview are developed using the Web Ontology Language (OWL) [19] and contain rich axiomatizations. Using the many axioms generated in the ontologies, the OWL reasoning can be used to support the inference of classification and direct information queries in the ontology. In addition, the construction of the classification hierarchy based on OWL reasoning can also be used to support enriched SPARQL queries [15] over the ontology information stored in an RDF triple store.

Based on the OWL-based classification and reasoning features, the CLO subset of LINCS cell lines was used for further analysis. The subset statistics was first generated and analyzed. Different types of LICNS cell line information were queried and studied. For example, the cell types of LINCS cell lines were studied based on the Cell Type Ontology (CL) [3], and the diseases modeled by the LINCS cell lines were studied based on the Disease Ontology [20] hierarchical structure information.

## Results

### LINCS cell line information extraction and mapping from different resources

**Fig. 2:**
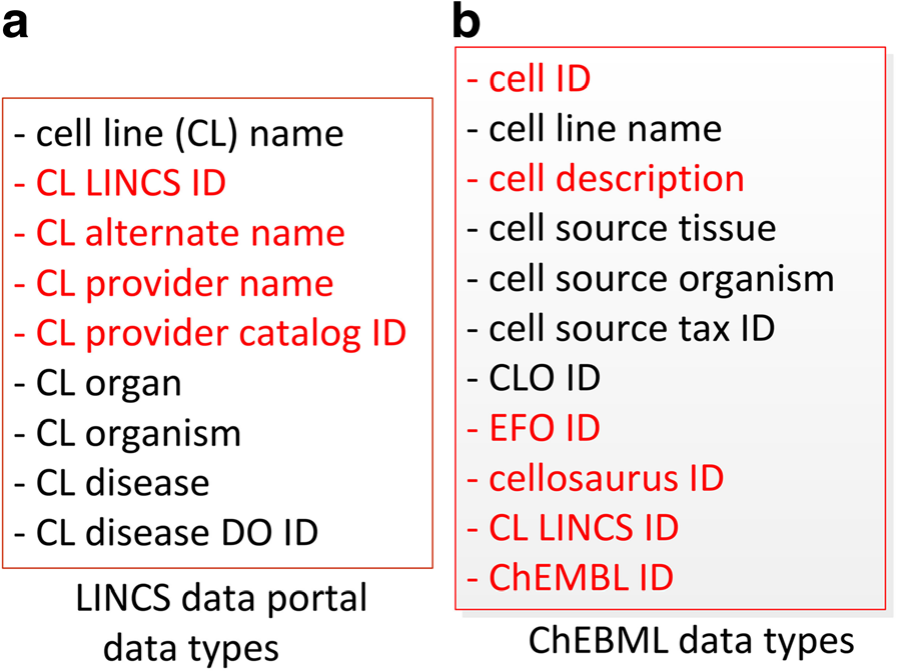
Cell line-related data types of the data downloaded from LINCS Data Portal and ChEMBL. **a** Data types from LINCS Data Portal. **b** Data types from ChEMBL. Red-highlighted items (e.g., ChEMBL ID) were not covered in CLO, which were added later to CLO in this study

As of June 15, 2017, 1097 cell lines were extracted from the LINCS Data Portal. Out of these LINCS cell lines, 794 cell lines could be directly mapped to CLO based on exact name matching and manual verification. A cell line may have different synonyms. The name matching used the default label and different synonyms for lexical mapping between these two resources. The data types related to these cell lines are listed in Fig. 2a. Meanwhile, the ChEMBL database included 637 cell line entries that have LINCS IDs. Out of these cell lines, 451 cell lines also have CLO_IDs, and 51 out of the remaining 186 cell lines could be mapped to CLO using name matching. The data types available related to these cell lines in ChEMBL are shown in Fig. 2b.

Among the 1097 LINCS cell lines each with a unique LINCS cell line ID (e.g., LCL-1512 for HeLa cell), 466 had ChEMBL, LINCS, and CLO IDs, 279 had both LINCS and CLO IDs, and 352 LINCS cell lines did not have any CLO IDs.

Note that sometimes one LINCS ID maps to multiple CLO IDs. For example, the Hep G2 cell line (http://www.atcc.org/products/all/HB-8065.aspx) has the LINCS ID LCL-1925, and it is mapped to three CLO IDs: CLO_0003704 (term label: Hep G2 cell), CLO_0050856 (label: RCB1648 cell), and CLO_0050858 (RCB1886 cell). Although they are all for Hep G2 cell based on their annotations,CLO_0003704 was the originally assigned based on an annotation from the ATCC cell line repository, and the other two come from the Japan RIKEN cell line bank with different registry information. In current CLO, we assert the two Japan cell bank cell line cell terms as subclasses of the CLO_0003704 with the consideration that the two Japan cell bank cell line cell types may have genetic variations given their long time of passages. In this case, all the three CLO cell line cell terms have the same LINCS cell line ID, which is defined using an annotation property ‘Cell line LINCS ID’.

### CLO modeling and design pattern generation

**Fig. 3:**
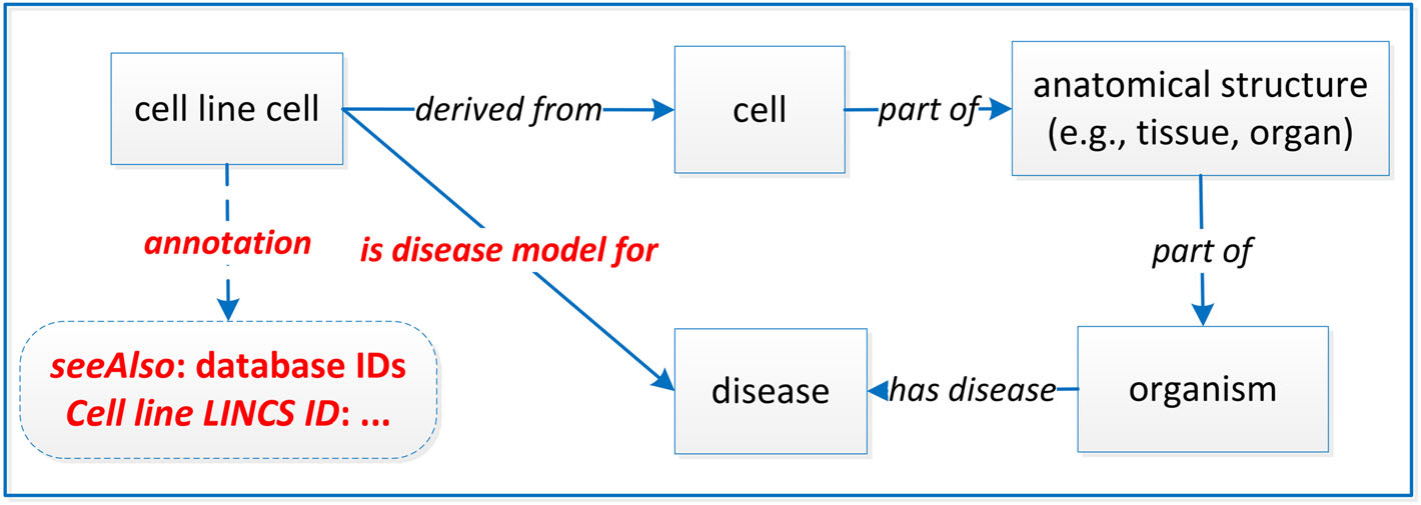
Basic CLO design pattern model for integrating LINCS cell line information from LINCS and ChEMBL

In CLO, the basic unit for representing a cell line is the term ‘cell line cell’, which is defined as “a cultured cell that is part of a cell line - a stable and homogeneous population of cells with a common biological origin and propagation history in culture” [8]. As shown in Fig. 1, the new cell line information identified from the LINCS project and ChEMBL database is of different types of names/description and data resource IDs. Such information can be effectively represented as specific annotation types. The strategy is reflected in a simple CLO design pattern model (Fig. 3), which was generated based on the general CLO design pattern reported in the original CLO paper [8].

For example, for the HeLa cell (CLO_0003684), based on the updated design pattern, we added the following information to CLO: ‘*Cell line LINCS ID*: LCL-1512’ and ‘*seeAlso*: EFO: EFO_0001185; CHEMBL: CHEMBL3308376; CVCL: CVCL_0030’.

Most LINCS cell lines were originally derived from human patients with some specific cancer diseases, and many of these diseases were not included in CLO. In this study, we imported corresponding disease terms from the Human Disease Ontology (DOID) [20]. To represent the relation between a cell line cell and a disease, we generated a new object property called ‘is disease model for’ (CLO_0000179). For example, for the HeLa cell, an OWL SubClassOf axiom was generated to represent its usage in studying cervical adenocarcinoma:

‘HeLa cell’ (CLO_0003684)*: ‘is disease model for’ some ‘cervical adenocarcinoma’*

It is noted that in CLO, the new object property ‘is disease model for’ is equivalent to the original object property ‘is model for’, an object property originated by the EBI cell line project (http://www.ebi.ac.uk/cellline#is_model_for) [8]. The EBI cell line project relation is obsolete. Replacing the obsolete legacy object property ‘is model for’ with the new CLO relation supports the ontology updating and standardization.

**Fig. 4:**
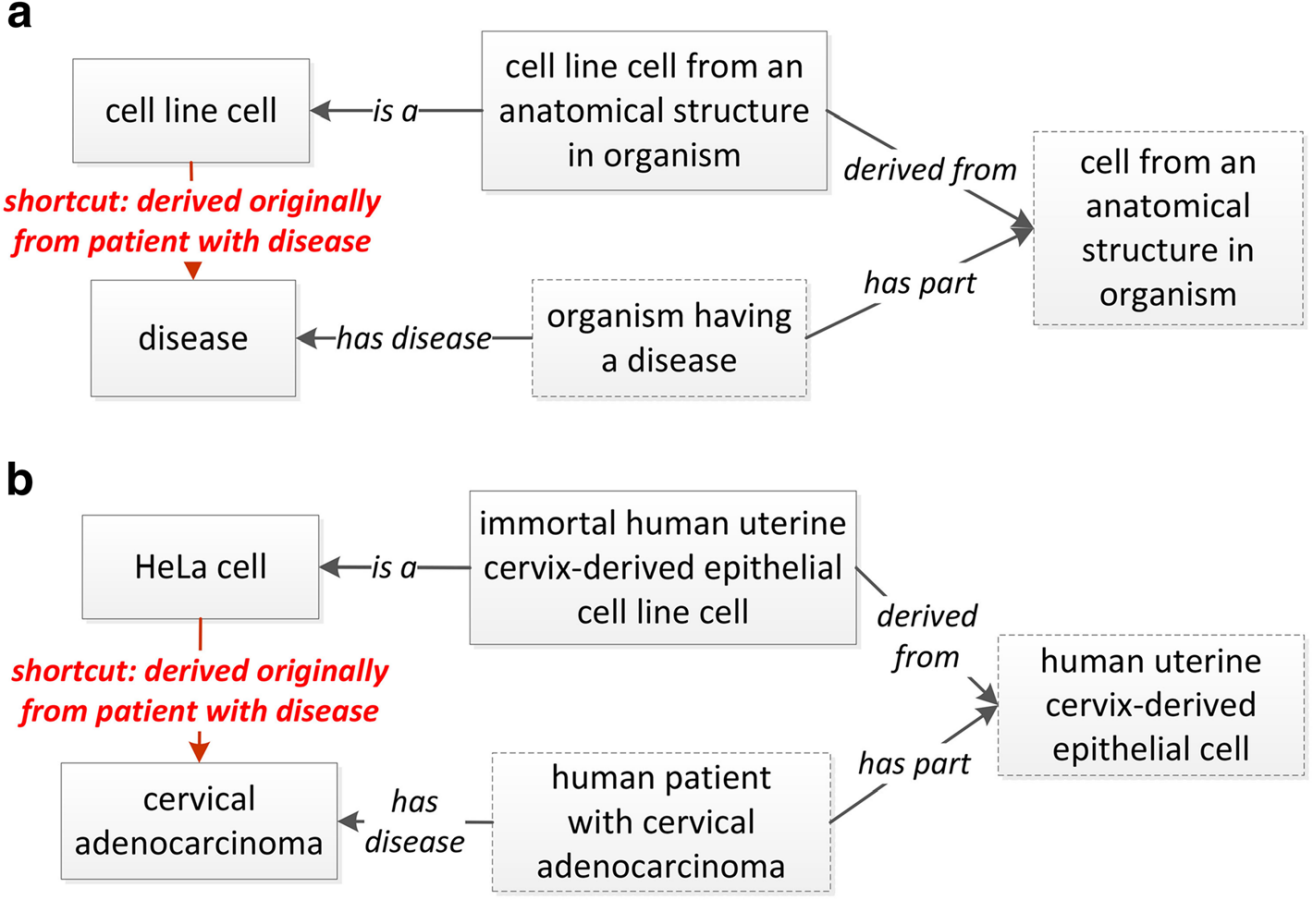
CLO design pattern model for using the new shortcut relation ‘derives originally from patient having disease’. **a** General design pattern; **b** an example to illustrate the design pattern. The shortcut relation makes it more efficient to represent the relation between a cell line cell and a disease when the parent term of the cell line cell includes sufficient information about the cell type and tissue/organ. In this illustration, the classes as shown in the dotted boxes are redundant and are not needed

The direct link between a disease and a cell line as a model to study the disease is required by LINCS data structure. In addition to this direct link, CLO also presents the origination of a cell line from a formalized ontological representation. The disease that is modeled by a cell line is often the disease of the particular human patient from whom the first passage of the cell line was originally generated. For example, the HeLa cell’s origin was the cervical adenocarcinoma cells separated from a cervical cancer patient, an African American woman in 1951 [21]. To represent the relation between the disease and the patient (original source for the cell line), the Fig. 4 design pattern was applied. For example, the following OWL SubClassOf axiom represents a human-cell relation for the HeLa cell in CLO:

‘HeLa cell’: *‘derives from’ some (‘epithelial cell’ and (part_of some (‘uterine cervix’ and (part_of some (‘Homo sapiens’ and (‘has disease’ some ‘cervical adenocarcinoma’)))))*

It is noted that HeLa cell is listed in CLO as a subclass of ‘immortal human uterine cervix-derived epithelial cell line cell’ (CLO_0000636), where the relation between human and the cell is clearly stated.

It is also noted from the above axiom that the long chain of axiom (i.e., cell line cell – cell type – tissue – organ – organism – disease) shown above becomes technically inefficient to query the relation between the cell line cell and the disease ‘cervical adenocarcinoma’. A shortcut relation (or object property) is a relation that is used to replace the usage of a chain of multiple relations and classes to represent the complex relations between two classes. Therefore, a new shortcut relation (or called object property) ‘derives originally from patient having disease’ (Fig. 4) was generated to directly link the cell line cell and disease as shown in the following OWL SubClassOf axiom:

‘HeLa cell’: *‘derives originally from patient having disease’ some ‘cervical adenocarcinoma’*

Although ‘is disease model for’ and ‘derives originally from patient having disease’ both represent a relation between a cell line cell and a disease, these two relations differ in their meaning. The shortcut relation ‘derives originally from patient having disease’ represents that the cell line cell was originally derived from a patient with a specific disease. The relation ‘is disease model for’ indicates that the cell line can be used to study a specific disease, and the disease can but does not have to, be the same as the disease of the patient from whom the cell line cell was derived. For example, HeLa cell can be used as a cell line model to study cervical adenocarcinoma, but it can also be used to study many other diseases such as polio and NewCastle Disease [21, 22].

### CLO modeling of cell features under regular cell culture conditions

In this study, we used the MCF 10A cell line cell as an example to show how CLO can be used to model cell features.

MCF 10A cell line cell is non-tumorigenic [23]. CLO represents such knowledge using the following OWL SubClassOf axiom:

‘MCF 10A’: *has_qality some non-tumorigenic*

where the *non-tumorigenic* is represented as a quality, and the relation between MCF 10A cell line cell and the quality can be represented using the object property *has_quality*.

MCF 10A cell line cells exhibit three dimensional growth in collagen and form domes in confluent cultures [23]. We can use the following OWL SubClassOf axiom to represent such knowledge:

‘MCF 10A’: *‘participates in’ some (‘three dimensional cell growth’ and (‘has participant’ some collagen)*

In this case, *‘three dimensional cell growth’* (CLO_0037311) is a process, and both MCF 10A cell line cell and collagen are participants of such a process. Since GO does not have such a *‘three dimensional cell growth’* term, we generated the term using a tentative CLO ID (CLO_0037311) and listed it as a subclass of the ‘cell growth’. Here the collagen is a component needed for the three dimensional cell growth. Collagen (CHEBI_3815) is a group of fibrous proteins of very high tensile strength that form the main component of connective tissue in animals.

### CLO modeling of cellular responses to special agent treatments

How to represent a cellular response of a cell line cell to a specific agent that is not part of regular cell culture media? Here we again use MCF 10A cell line cell response modeling as an example study.

It is known that MCF 10A mammary epithelial cells undergo apoptosis following actin depolymerization. The MCF 10A response can be represented in the following OWL SubClassOf axiom:

‘MCF 10A’: *‘participates in’ some (‘apoptotic process’ and ‘preceded by’ some ‘actin depolymerization’ and (‘induced by cell culture reagent’ some latrunculin-A))*

In this case, *apoptotic process* is represented as a GO term (GO_0006915). This process in MCF10A cells occurs after actin depolymerization (GO_0030042) is induced by a cell culture reagent Latrunculin A (CHEBI_69136), a bicyclic macrolide natural product consisting of a 16-membered bicyclic lactone attached to the rare 2-thiazolidinone moiety [24].

Sometimes, cell line cells were genetically engineered to generate a new cell line by a transfection process. Basically, a transfection process deliberately introduces naked or purified nucleic acids into eukaryotic cells such as cell line cells. For example, MCF10A-Er-Src cell line cell is a MCF10A cell derived cell through transfection. As a result, MCF10A-Er-Src cell has the part of ER-Src, a derivative of the Src kinase onco-protein that is fused to the ligand-binding domain of the estrogen receptor (ER). It is clear that MCF10A-Er-Src cell line cell is not a subtype of MCF 10A cell. The transfection process makes the new cell a MCF 10A–derived cell type instead of a subtype of MCF 10A per se. Specifically, CLO represents the new MCF10A-Er-Src cell line cell formation as shown in the following OWL SubClassOf axiom:

‘MCF10A-Er-Src cell’: *‘is specified output of’ some (‘cell line cell transfection’ and (‘has specified input of’ some ‘MCF 10A cell’))*

### LINCS-CLOview: LINCS cell line subset of CLO

Based on the mapping and the design pattern models (Figs. 3 and 4), extra data available in the LINCS Data Portal and ChEMBL were integrated into to CLO. To improve the efficiency, a combination of manual annotation/edition and Ontorat [16]-assisted automated process was conducted.

The new information added to CLO includes two parts:

1. Existing 795 CLO cell line cell items were added with newly obtained data (Fig. 2), e.g., LINCS cell line IDs and disease information. All the disease information was mapped to the Human Disease Ontology (DOID) [20].
2. 352 LINCS cell lines unavailable in CLO were newly added to CLO. Each of these cell lines was assigned a new non-redundant CLO ID based on CLO cell line naming convention [8]. The parent terms of these newly added CLO cell lines were determined by the cell type, tissue, organ, and organism. All the cell lines were found to be derived from human. The diseases in the human patients were primary cancers. Three cell lines were derived from patients with benign tumors.

### LINCS-CLOview: LINCS cell line subset of CLO

A CLO subset of LINCS cell lines, abbreviated as LINCS-CLOview, was generated. The LINCS-CLOview can be considered as a “community view” [25] or a slim of the CLO’s implementation of LINCS cell lines for the LINCS research community. As of July 1, 2017, LINCS-CLOview contained 1924 terms, including 1825 classes, 25 object properties, 61 annotation properties, and 13 instances. These terms include 1315 cell line cell terms with CLO IDs. The other terms were imported from 17 other ontologies, for example, the Basic Formal Ontology (BFO) [26], the Cell Type Ontology (CL) [3], and the Ontology for Biomedical Investigations (OBI) [4]. The LINCS-CLOview source code is included in the master CLO GitHub website. The detailed statistics of LINCS-CLOview is available at: http://www.ontobee.org/ontostat/LINCS-CLOview.

As a subset of CLO, LINCS-CLOview has the same hierarchical structure and design patterns as the CLO. BFO is the top-level ontology with which CLO is aligned. Since BFO is also the top-level ontology for over 100 ontologies (e.g., CL and OBI), such an alignment makes LINCS-CLOview easily integrated with other ontologies, such as CL for cell types, and OBI for cell line related processes.

### SPARQL query of LINCS-CLOview information

**Fig. 5:**
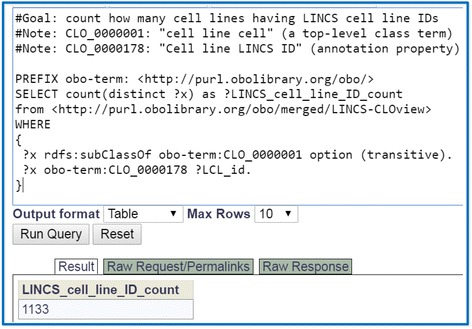
SPARQL query of the number of cell lines with LINCS ID annotation. The query was performed using Ontobee SPARQL (http://www.ontobee.org/sparql)

The Ontobee SPARQL web query program can be used to conveniently query detailed information in LINCS-CLOview. For example, Ontobee SPARQL was used to query the number of cell line cells that have the LINCS cell line IDs (i.e., LCL_xxxx) (Fig. 5). The script recursively queries all class terms under the branch of ‘cell line cell’ (CLO_0000001) in LINCS-CLOview, identifies those terms having the ‘Cell line LINCS ID’ (CLO_0000178), and counts the total number of these cell line cell terms. As shown in the figure, the total unique number of these LINCS cell line cells with LINCS cell line IDs in LINCS-CLOview (or CLO) is 1133. This number is greater than 1097 LINCS cell lines extracted from our processes, which is because one LINCS ID may sometimes be mapped to more than one cell line in CLO as indicated at the beginning of the Results section. If we do not consider the LINCS cell line IDs, we would get 1541 cell line cell terms under this cell line cell branch in the LINCS community view of the CLO. The difference between these two numbers reflects the fact that there are many intermediate-layer cell line cell terms between the LINCS cell lines (with LINCS IDs) and the ‘cell line cell’ (CLO_0000001) in the LINCS-CLOview.

In this study, different SPARQL scripts were developed and used to analyze the LINCS cell lines from various aspects. An example of such SPARQL analysis is illustrated in next section.

### Analysis of LINCS cell lines by querying LINCS-CLOview

With the availability of LINCS-CLOview, we were able to analyze LINCS cell lines from different aspects. The tools used in our analyses include SPARQL-based queries, Protégé OWL editor visualization, and Ontobee statistics display and queries. Below we describe our analyzed results from three main aspects: related diseases, cell types, and tissues/organs.

**Fig. 6:**
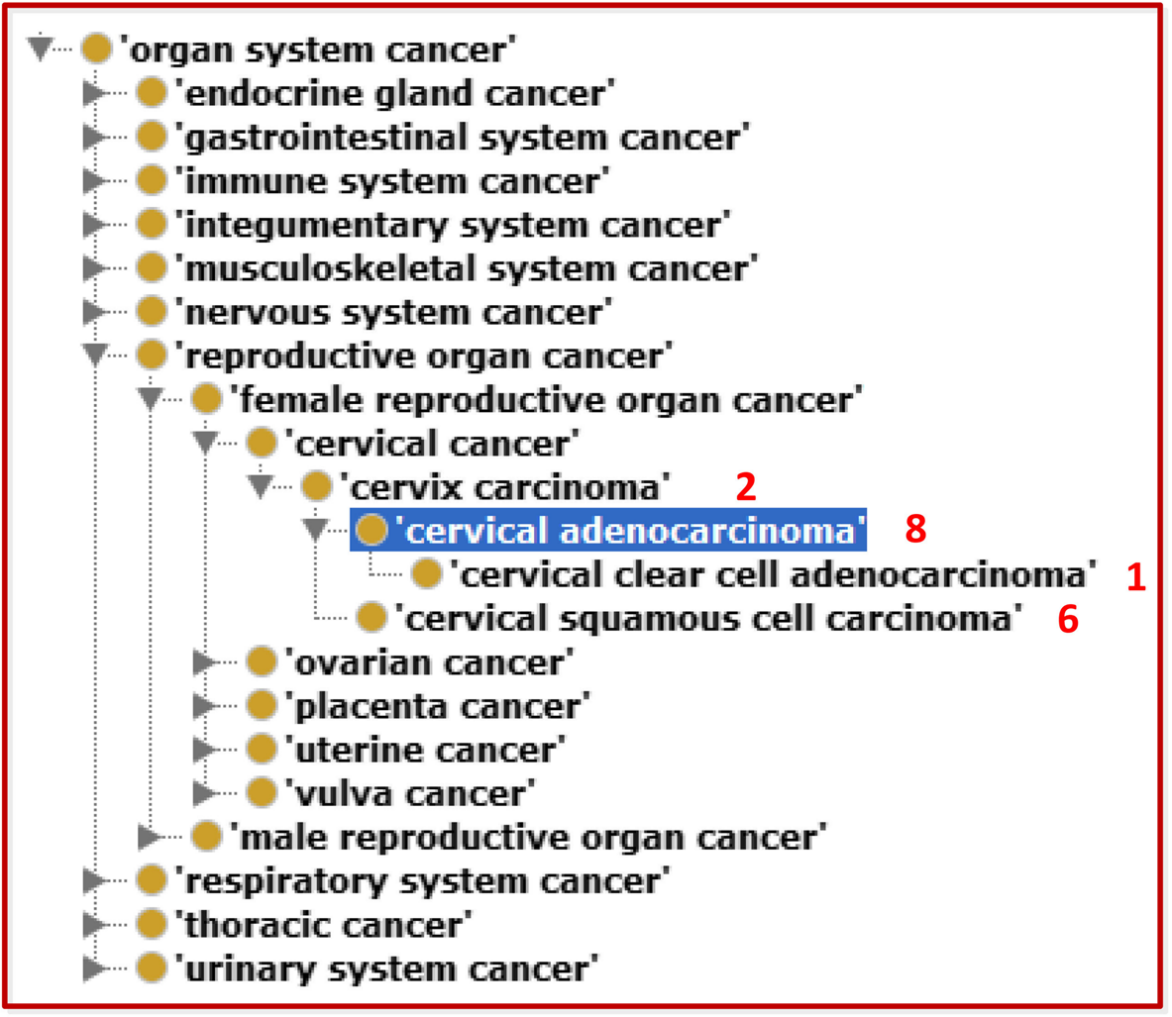
The DOID hierarchy of 121 diseases of patients from whom 1133 LINCS cell lines were derived. The red color numbers represent the number of LINCS cell lines that are associated with corresponding diseases

Our study found that LINCS cell lines are associated with 121 diseases. These 121 diseases include three benign neoplasms, i.e., breast fibrocystic disease (associated with MCF 10A and MCF 10F cells), kidney angiomyolipoma (associated with 621–101 cell), and male productive organ benign neoplasm (associated with BPH-1 cell). The other 118 diseases are various types of cancers. Fig. 6 is a hierarchical DOID structure of organ system cancers related to these LINCS cell lines.

The hierarchical structure of DOID (Fig. 6) helped the understanding of all the diseases associated with LINCS cell lines. For example, Fig. 6 demonstrates that 8 LINCS cell lines (e.g., HeLa cell) were derived from patients with cervical adenocarcinoma, 1 with cervical clear cell adenocarcinoma (a specific type of cervical adenocarcinoma), and 6 with cervical squamous cell carcinoma. These diseases all belong to cervix carcinoma. In addition, ‘cervix carcinoma’ is directly associated with 2 LINCS cell lines (i.e., C-33 A and C-4 II cell line cells). Therefore, if we plan to study the cellular signatures of cervix carcinoma, we would focus on these 17 cell lines instead of just 2 cell lines directly annotated as derived from a patient having cervix carcinoma.

**Fig. 7:**
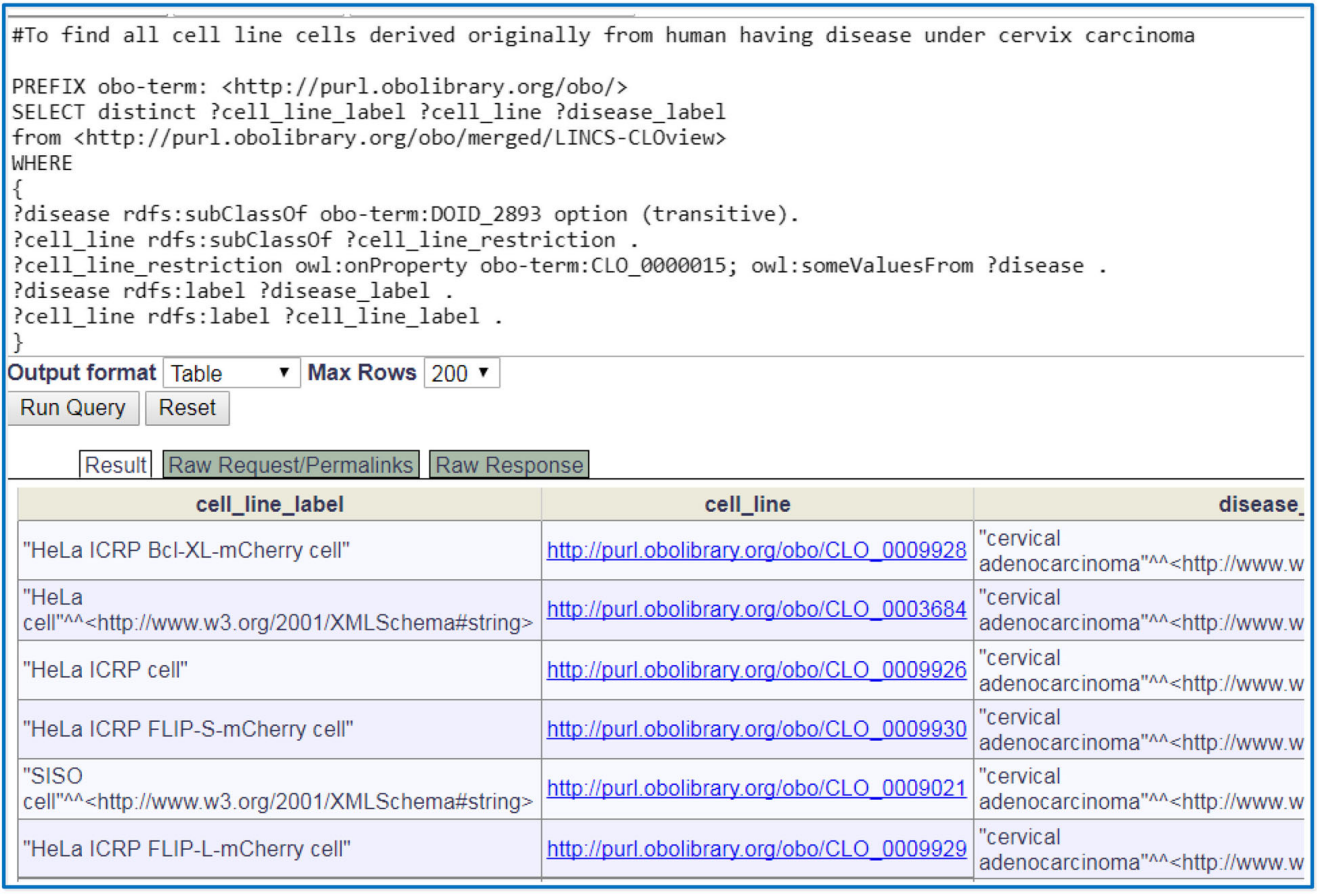
Identification of additional cell lines under cervix carcinoma by SPARQL querying LINCS-CLOview. Note that DOID_2893 is the class ‘cervix carcinoma’, and CLO_0000015 is the object property ‘derives originally from human having disease’. In total 15 cell line cells were identified. Here only 6 are shown up. The query was conducted using Ontobee SPARQL (http://www.ontobee.org/sparql)

To further illustrate the usage of LINCS-CLOview, we generated a SPARQL script that queries the cell lines originally derived from human patients having more specific disease names under cervix carcinoma (Fig. 7). Consistent with Fig. 6, our query identified 15 new cell line cell types (e.g., HeLa cell line cell) that belong to this category, and 5 identified cell line cell types are shown in Fig. 7.

**Fig. 8:**
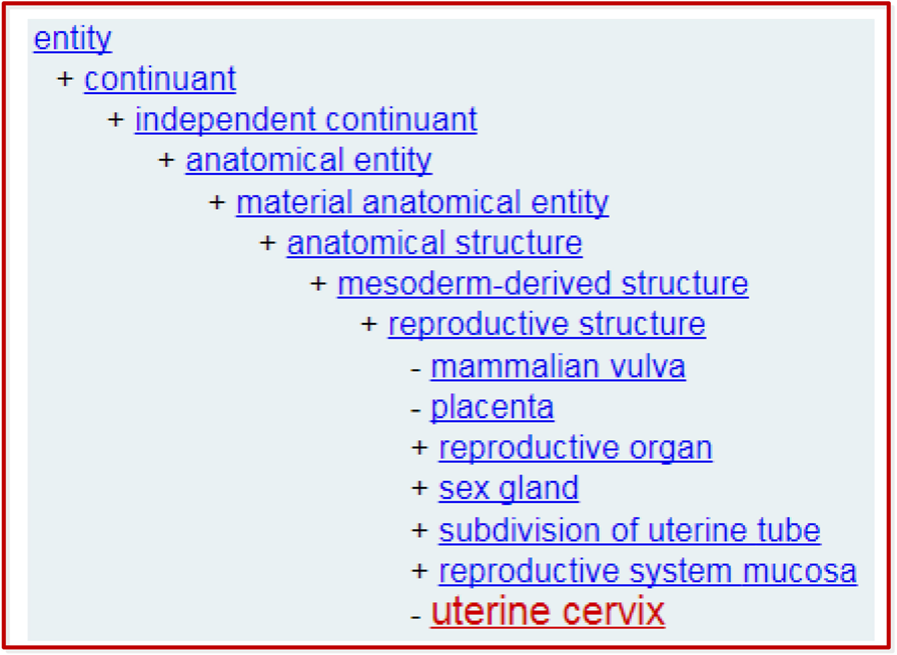
Part of the UBERON hierarchical structure in LINCS-CLOview. This structure shows the location of ‘uterine cervix’**.** This is a screenshot of the UBERON term in the LINCS-CLOview Ontobee web page

We also examined the tissue and organ types from which the LINCS cell lines were derived. In CLO, the multi-species anatomy ontology UBERON [27] is used to represent tissues and organs. In total 131 UBERON terms have been used in LINCS-CLOview to refer to various anatomic locations from which LINCS cell lines were derived. A part of the UBERON structure is illustrated in Fig. 8.

The cell types of LINCS cell lines were analyzed. The Cell Type Ontology (CL) [3] was used in CLO to demonstrate the cell types of different cell lines. In total, 43 CL cell types, such as epithelial cell, B cell, and T cell, are included in LINCS-CLOview. Each of these cell types is linked to different cell line cells or the parent terms of cell line cells. For a project to study cellular signatures related to a specific cell type, the LINCS-CLOview provides a feasible method to identify which cell line cells to use.

## Discussion

The contributions of this study are multiple. First of all, we systematically annotated the LINCS cell lines and integrated the information of the cell lines from different resources to CLO. Second, two more object properties, including ‘is disease model for’ and ‘derives originally from patient with disease’, were newly generated in CLO in order to use CLO to represent the information of LINCS cell lines. Third, using MCF 10A, we show how to use CLO to represent cell line cell features and a cellular response to an extracellular agent. Fourth, we generated the LINCS-CLOview, a CLO community view for the LINCS research community, which will serve as a CLO standardized module for LINCS data integration and coordination. Fifth, useful information about the LINCS cell lines was obtained via analyzing LINCS-CLOview. Given well-defined class hierarchy and axiom assertions and OWL reasoning, our analyses showed that ontology SPARQL was also able to query results for different use cases. This study is timely updating and implementation of CLO for enhanced cell line data integration and analysis features.

We have for the first time showed in this study how to use CLO to represent specific cellular responses to agents such as collagen, Latrunculin A, or transfection agents. Such treatments make the cells form 3D growth, apoptosis, or a new cell line cell type. Each cellular process is represented separately based on its own characteristics. More specifically, for each process, we typically use the pattern of ‘participates in’ some specific process which is either a term obtained from an existing ontology such as GO, or a term generated in CLO if such a process term cannot be found from any existing community-based ontology. After such modeling discussed and agreed by the manuscript reviewers and presentation audience, we plan to extend such design patterns to represent cellular responses of other CLO cell line cells.

To our knowledge, this article is the first report of implementing CLO by developing a CLO community view (or slim) to serve a specific community, in this case, the LINCS research community. Since tens of thousands of cell lines have been represented in CLO, it is not efficient to use the whole CLO for LINCS cell line related research. The generation of LINCS-CLOview in this study allows the standardization and modularization of the LINCS cell lines, which facilitates the better analysis and reuse of the LINCS cell line information. Such a strategy can also be used to represent and study cell lines used by other communities.

One advantage of this CLO-based LINCS cell line representation is to bring together different cell line resources (e.g., LINCS, ChEMBL, and Cellosaurus) with the framework of CLO. Our integration of LINCS cell lines and CLO ensures the consistency and integrity of the cell line data across multiple resources. In many cases, a key challenge of systematically studying diverse systems biology signature data such as LINCS is the difficulty in integration of different data types and information related to various cell lines. LINCS-CLOview provides a cell line-oriented semantic framework and foundation for integrating different cell line study results and supporting systematical analysis of cellular signatures and network studies.

Our future work will include different directions. One major direction is to apply the ontological representation of LINCS cell lines in LINCS-CLOview to systematically study cellular molecular markers. For example, we can study cellular markers based on different criteria such as disease, cell type, tissue, and organ. As illustrated in Figs. 6 and 7, if we want to study cervix carcinoma, we can easily identify 17 related cell lines. The hierarchical structures of different types of entities are useful since you can often find more information. For example, based on the Fig. 6 hierarchy, there are clearly more than 2 cell lines that are related to cervix carcinoma. Another possible future work is to apply LINCS-CLOview to map cell lines to specific experimental LINCS datasets or specific project(s) and identify scientific insights that associate different types of cell line cells with experimental conditions. We also plan to study cell line cell-specific gene/protein interactions and pathways and compare them based on different types of cell line classifications.

As a community-based ontology, the CLO team is also collaborating with other research projects that heavily use cell lines, such as the European Bioinformatics Institute (EBI) that uses many cell lines in their EBI projects. EBI uses the Experimental Factor Ontology (EFO) to represent various experimental factors such as cell line cells. We are now teaming with EBI to synchronize the cell line cells in EFO and CLO. We are also working with the China Cell Line Bank group to add their cell lines and their specific needs to CLO. Such activities will make CLO a more community-based centralized ontology source for cell line cell representation, supporting integrative cellular research to solve different scientific questions.

## Conclusions

In summary, we updated CLO by incorporating the information of all identified LINCS cell lines and used CLO to model cellular responses to different cell culture growth conditions and perturbations. LINCS-CLOview, a CLO subset of the LINCS cell line information, was generated and analyzed to better understand LINCS cell line information.

## Abbreviations

ATCC: American Type Culture Collection
BAO: BioAssay Ontology
CL: Cell Ontology
CLO: Cell Line Ontology
EBI: European Bioinformatics Institute
EFO: Experimental Factor Ontology
LINCS: NIH Common Fund Library of Integrated Network-based Cellular Signatures
NIH: National Institutes of Health
OBI: Ontology for Biomedical Investigations (OBI)
OBO: Open Biological and Biomedical Ontologies
